# COVID-19 Vaccine Hesitancy Among Pregnant Women

**DOI:** 10.7759/cureus.41126

**Published:** 2023-06-29

**Authors:** Manal Alkhalifah, Noara AlHusseini, John McGhee

**Affiliations:** 1 Public Health, Alfaisal University, Riyadh, SAU; 2 Public Health, Center for Improving Value in Health Care, Riyadh, SAU

**Keywords:** covid-19, vaccine efficacy, high-risk population, vaccine acceptance, hesitancy

## Abstract

Background

COVID-19 has struck the world severely and caused much damage, losses, and a massive impact on different aspects of life. It is an airborne disease that spreads rapidly among populations and can cause severe illness or death. The rapid nature of its spread led to significant challenges to control it. With the introduction of vaccines, strategies need to be developed to prioritize high-risk populations to lower complication rates, hospitalization, and death. Pregnant women are considered a group of high-risk populations. Misinformation about the vaccination efficacy or side effects contributed to general hesitancy, especially among pregnant women.

Purpose

This study aims to describe the drivers of COVID-19 vaccine hesitancy among pregnant women in Saudi Arabia.

Methodology

This is a cross-sectional study among pregnant women in the OB/Gyn clinic in King Abdulaziz Medical City, Riyadh, Ministry of National Guard Health Affairs (MNG-HA), using an online survey. Descriptive statistics (univariate analysis) was used to examine the population characteristics. The Chi-square test was used for categorical variables, and t-test for continuous variables. Further, we used the logistics regression model (multivariate analysis), adjusted for potential confounders, to examine factors associated with women's hesitancy to take the COVID-19 vaccine. All statistical tests were two-sided, and findings were considered statistically significant at p < 0.05. All analyses were conducted using SAS statistical software version 9.4 (SAS Institute Inc., Cary, NC).

Result

The study included 303 pregnant women. Nearly half of the respondents had their vaccine during their pregnancy (42.24%), believing that the current vaccines' effectiveness for the coronavirus is good (41.25%). More than 73% of participants have received their COVID-19 vaccine before pregnancy. The mean hesitancy and anxiety score was 2 (agree), which concluded that the respondents were hesitant and anxious to receive the COVID-19 vaccine.

Conclusion

The study showed a significant correlation between pregnant women's worries and the intention to take the vaccine. The concerns were mainly about the impact of the vaccine on themselves, their babies, and the pregnancy.

## Introduction

COVID-19 global and local precaution regulations

The fast-spreading COVID-19 pandemic has hit countries around the world. Governments implemented different measures to contain the spread of the disease. Containment measures varied between the closure of educational and entertainment institutions, lockdowns, and restricted physical distance measures. Researchers have studied the effectiveness of these containment measures across different countries. The study showed a significant reduction in the spread of disease due to implementing containment measures [1].

Impact of COVID-19 on mental health, social life, and life expectancy

The continuous global pandemic of COVID-19 will have a long-term impact. It severely impacted social life and social interactions [2]. This pandemic led to increased mental health problems, anxiety, and depression. It resulted in a sizeable number of deaths around the world. The total number of global deaths because of COVID-19 was 819,612 in August 2020, which is growing to date [3]. With the increasing mortality, life expectancy will be affected across countries. A study in 2020 estimated the potential direct impact of COVID-19 on life expectancy. The result showed that at 10% infection prevalence, over one year of life expectancy was lost, and at 50%, five years were lost [3].

COVID-19 vaccines

COVID-19 vaccine development started in 2020, and in 2021 it was deployed in many countries. Several vaccines proved their efficiency and were approved in different countries. Some vaccines' effectiveness reached up to 94%, reducing the risk of infection. However, two main hurdles were faced in deploying vaccines. The first was vaccine hesitancy, and the second was access in some countries [4].

With the enormous economic damage and collateral life losses, the economic value of the COVID-19 vaccine is self-evident. The health results of the vaccine deployment are promising to end the pandemic. While increasing competition among manufacturers, it is recommended to have value-based pricing to incentivize efficacy and ensure affordability [5].

COVID-19-approved vaccines in Saudi Arabia

The population of Saudi Arabia is about 34 million. A total of 67% of the population needs to be vaccinated to reach herd immunity [6]. Besides the different containment measures to limit the spread of the COVID-19 pandemic, scientists and pharmaceutical companies were deploying clinical trials and developing vaccines to improve prevention. Saudi Arabia was one of the beginners to approve using the new modified RNA vaccine produced by Pfizer‑BioNTech with an efficacy of 95%. Moderna produced another RNA vaccine approved for use in Saudi Arabia with an efficacy of 94%. In December 2020, Saudi Arabia received the first shipment of the COVID-19 vaccine and started a broad vaccination campaign immediately for everyone for free. Oxford-AstraZeneca was one of the approved vaccines in Saudi Arabia and has proved efficacy in reducing disease and mortality [7]. Upon the deployment of the vaccination in Saudi Arabia, a special COVID-19 vaccine committee was established in tandem with a technical advisory group. Strategies and process plans were developed to ensure timely distribution of the vaccination across regions. Priority population groups were identified to ensure equity in distribution. In order to guarantee a standardized and high-quality immunization process, the Ministry of Health developed a blueprint for all entities to use [8]. Vaccination was initiated in a phased approach, targeting high-risk populations and healthcare professionals first [9].

COVID-19 vaccines' value and efficacy

The COVID-19 vaccination is the most effective approach to preventing the disease's spread. It saves lives and reduces the productivity loss caused by lockdowns during the pandemic. Cost and benefit analysis has shown that one dollar invested in the vaccine granted benefits from $13-28 USD in return [10]. The COVID-19 vaccine has been evaluated for over a year and has proved efficacy and safety. The Specialized Scientific Committee in Saudi Arabia has recommended the COVID-19 vaccine for pregnant women for its safety for both the mother and the fetus. It was approved for use in Saudi for pregnant women in April 2021 [11].

COVID-19 vaccine hesitancy among pregnant women

One of the high-risk groups for being infected with COVID-19 is pregnant women. During pregnancy, physiological changes may affect the immune system and other body systems, contributing to disease progression in infected women [12]. Hesitancy among pregnant women has been studied in different countries. The findings showed that the vaccination rate is lower among pregnant women than non-pregnant women in the United States. Several factors contributed to the low vaccine coverage among pregnant women, such as lack of safety information about the vaccine during pregnancy, culture, hesitancy, and access. Therefore, timely and accurate information about vaccine safety during pregnancy is critical to increasing acceptance of the COVID-19 vaccine among this high-risk group [13]. Pregnant women tend to be hesitant about vaccination. The rate of maternal influenza vaccine uptake is low in European countries. A systematic review was done to understand the determinant of influenza vaccine hesitancy in Europe. The highest factor attributing to hesitancy was the psychological aspects like worries about safety and resultant risks to the mother and child. Other factors were doubts regarding the vaccine's effectiveness and inadequate vaccine knowledge [14]. This study will describe the drivers of vaccine hesitancy among pregnant women in Saudi Arabia via a sample from the National Guard Hospital.

## Materials and methods

This research is a cross-sectional study among pregnant women following up at the OB/Gyn clinic at King Abdulaziz Medical City, Riyadh, Ministry of National Guard Health Affairs (MNG-HA). An online survey via Google Forms was created and distributed via the barcode. We received 303 responses. The survey evaluated socioeconomic attributes, vaccination history, and attitude toward COVID-19 vaccination. Data collection was done from December 8, 2021 to March 7, 2022, by distributing a printed barcode of the survey on the pregnant women visiting the OB/Gyn clinic at MNG-HA in the waiting area.

The survey was taken from a similar study done in Ankara, Turkey, by the Department of Obstetrics and Gynaecology, Ministry of Health, Ankara City Hospital, after taking the permission of one of the authors Dr. Şule. It was modified to serve our local study's purpose and context and was pre-tested and validated by eight pregnant women. The survey was distributed in the Arabic language after word-by-word official translation to Arabic to achieve face validity.

The survey has three main sections. The first section includes the sociodemographic data of the participant, like age, residence, education status, and career. The second covers the participants' health status and information about the current pregnancy. Finally, the third part focuses mainly on vaccination knowledge and information and the reasons for hesitancy or rejection.

We have added a Cambridge worry scale, a self-administered questionnaire to assess the content and extent of pregnancy-related worries. It contains statements about issues that might be a reason for concern for a pregnant lady. Each statement is scored on a six-point Likert-type scale ranging from not a worry (0) to major worry (5).

The sample size was estimated by having the number of pregnant patients visiting the OB/GYN clinics at MNG-HA. With a two-sided alpha of 0.05 and a power of 0.8, the sample size was calculated to be 139, but we were able to include 303 participants. Proportional sampling was based on the prevalence of pregnancy in that clinic. 

The survey was anonymous, with no patient identifications. All data was kept secure within MNG-HA premises. Participation was optional, and the study's objective was stated clearly. Filling out the survey was construed as consent to participate. The investigator and the team had no conflict of interest. Approval had been obtained from the Institutional Review Board at King Abdullah International Medical Research Centre KAIMRC, reference number RYD-21-419812-176357.

Inclusion criteria

All pregnant women following up at the National Guard Hospital OB/GYN clinic.

Exclusion criteria

Non-pregnant women.

Statistical analyses

Descriptive statistics (univariate analysis) was used to describe the characteristics of the population, the Chi-square test for categorical variables, and the t-test for continuous variables. To examine the mean score of hesitancy, anxiety, and knowledge among the respondents, we used Generalized Linear Model (GLM). Assumptions of homogeneity and independence of residuals were assessed and met. Further, we used the logistics regression model (multivariate analysis), adjusted for potential confounders (all demographic variables shown in Table 1), to examine factors associated with women's hesitancy to take the COVID-19 vaccine. All statistical tests were two-sided, and findings were considered statistically significant at p < 0.05. All analyses were conducted using SAS statistical software version 9.4 (SAS Institute Inc., Cary, NC, USA).

## Results

The study included 303 pregnant women, slightly over half of them in the age group 20-30 (50.5%), and more than two-thirds of the respondents reported employment status as a housewife (70.96%). More than half of the population reported holding an undergrad degree (59.08%) and not having an income (51.18%). The respondents were predominately residing in the central region of Saudi Arabia (92.07%) (Table 1). Nearly one-third of the participants reported that this was their first pregnancy (30.03%). Approximately half of the population had one or two people in the household and school-aged children (40.92% and 45.54%, respectively). A large percentage of the respondents were at 25-40 weeks gestational age (79.87%), not at high-risk pregnancy (50.83%), did not have chronic conditions (69.97%), had no close contact with COVID-19-infected individuals (88.78%), have received their vaccines (73.6%), and have heard of COVID-19 vaccine before (90.43%). Nearly half of the respondents had their vaccine during their pregnancy (42.24%), believing that the current vaccines' effectiveness for the coronavirus is good (41.25%). The mean hesitancy and anxiety score was 2 (agree), which concluded that the respondents were hesitant and anxious to receive the COVID-19 vaccine (Table 2). The respondents had a mean score of 2 (neutral) regarding precautions and knowledge. The respondents neither agreed nor disagreed that they had sufficient precautions and knowledge about the COVID-19 vaccines.

**Table 1:** Demographic characteristics of participants in the study.

	Total N (%)
Total	303 (100)
Age	
20-30	153 (50.5)
31-40	132 (43.56)
41-50	18 (5.94)
Employment status	
Employed in governmental sector	53 (17.49)
Employed in private sector	23 (7.59)
Housewife	215 (70.96)
Other	12 (3.96)
Education attainment	
High school or less	115 (37.95)
Undergraduate education	179 (59.08)
Graduate education	9 (2.97)
What is your monthly income	
9,999 Saudi Riyal or less	31 (10.23)
10.000-19.999 Saudi Riyal	4 (1.32)
20.000 Saudi Riyal or more	32 (10.56)
I do not have a monthly income	157 (51.18)
I prefer not to answer	79 (26.07)
Region of residence	
Central Region	279 (92.07)
Eastern Region	8 (2.64)
Northern/south and western Regions	16 (5.28)
1 Chi-square test and Fisher's exact test	

**Table 2:** Pregnancy characteristics, vaccine knowledge, and worry scale of participants in the study.

	Total N (%)	P-value ^1^
Is this the first pregnancy?		< 0.0001
Yes	91 (30.03)	
No	212 (69.97)	
Number of people in the household		
One or two	124 (40.92)	
Three or four	94 (31.02)	
More than four	85 (28.05)	
Number of school-age children		
One or two	138 (45.54)	<0.0001
Three or four	56 (18.48)	
More than four	24 (7.92)	
I don’t have children going to school	85 (28.05)	
Gestational age in weeks		<0.0001
12-24 weeks	43 (14.19)	
25-40 weeks	242 (79.87)	
Less than 12 weeks	18 (5.94)	
Is this considered a high-risk pregnancy?		<0.0001
Yes	42 (13.86)	
No	154 (50.83)	
I don’t know	107 (35.31)	
Do you have any chronic conditions?		
Yes	84 (27.72)	< 0.0001
No	212 (69.97)	
I don’t know	7 (2.31)	
If the answer is yes, what condition/s do you have?		<0.0001
Diabetes	31 (10.23)	
Hypertension/Heart disease/High cholesterol	5 (1.65)	
Hypothyroidism/Hyperthyroidism	11 (3.63)	
Other	45 (14.85)	
No	188 (62.04)	
Did you have close contact with a COVID-19-positive person?		< .0001
Yes	34 (11.22)	
No	269 (88.78)	
Did you have COVID-19 in this pregnancy?		< 0.0001
Yes	33 (10.89)	
No	270 (89.11)	
Have you taken COVID-19 vaccine?		< 0.0001
No, I haven't received the vaccine, and don't want to have it	12 (3.96)	
No, I haven't received the vaccine, but I want to have it	21 (6.93)	
No, I haven’t received the vaccine	47 (15.51)	
Yes, I have received the vaccine	223 (73.6)	
If the answer is yes, when did you take your COVID-19 vaccine?		0.03
Before pregnancy	97 (32.01)	
During pregnancy	128 (42.24)	
Not mentioned	78 (25.74)	
Have you heard of COVID-19 vaccine before?		<0.001
Yes	274 (90.43)	
No	29 (9.57)	
The effectiveness of the current vaccines for the coronavirus is good	
High	125 (41.25)	
Moderate	90 (29.70)	
Low	9 (2.97)	
I don’t know	79 (26.07)	
Hesitancy scores mean (SD) 2		2 (1)
knowledge/precautions score mean (SD)		3 (1.3)
Anxiety scale mean (SD)		2 (1.55)
1 chi-square test and fisher exact test 2 mean and standard deviation (score range 0 to 5) Strongly disagree=5 Disagree = 4 Neutral =3 Agree=2 Strongly agree=1		

Nearly half of the cohort was 20-30 years old (50.5%), following the age group of 31-40 (43.56%). After examining the mean score with age groups, we found no significant association between the mean score and the different age groups. As for the larger age group 20-30 years old (estimate 0.08, 0.34, and -0.33; p-value 0.72, 0.22, and 0.39 respectively), no statistically significant association was found between the age group 20-30 and the age group 41-50 in terms of all scores. Among women who reported the risk of pregnancy, those who reported that they were at high risk had a higher mean anxiety score than those who reported no high-risk pregnancy (estimate 0.07; p-value 0.0009). There was a statistically significant association between anxiety score and pregnancy risk. In the multiple regression model adjusted for demographics, there was a significant positive association between the behavior of not having the vaccine and the mean anxiety score (estimate (β) 0.71, 0.99, and 0.63; p-value 0.02, 0.04, and 0.009, respectively). Those who have not received the vaccine but want to have it had the highest means of anxiety score compared to those who received it (estimate (β) 0.99; p-value 0.04). While those who have not received the vaccine but want to have it had an anxiety score of an average of 0.99 points greater than those who received the vaccine.

## Discussion

To our knowledge, this is the first study in Saudi Arabia investigating the factors of COVID-19 vaccine hesitancy among pregnant women. We found that 74% of the study population received the vaccine during or before the pregnancy. Although most of the pregnant women in our study received the vaccine, the worry/anxiety score about taking the vaccine was statistically significant. Women who did not receive the vaccine but were planning to take it have a higher anxiety score than those who did. Our study showed lower percentages than a study conducted in Egypt regarding the people's belief of getting infected after having the COVID-19 vaccine and regarding herd immunity. Respectively, 34% of the population believes that the COVID-19 vaccine might cause COVID-19 infection, and 44% believe that herd immunity is enough to protect everyone from the coronavirus. In contrast, in a study in Egypt, more than 86% of the study population believed that people could get infected even if vaccinated, and 62% believed that herd immunity from being infected is enough. We found in our study similar factors of hesitancy toward the COVID-19 vaccine with other studies. However, our identified factors varied between the possibility of harming the baby, the possibility of causing pregnancy complications, the safety of the vaccine during pregnancy, the chance of harming the pregnant woman, the potential of causing stillbirth, the possibility of causing genetic mutation, and the possibility of causing COVID-19 infection. The shared factors with other studies were side effects, perception of vaccine efficacy, perception of vaccine safety, and finally, perception of fake COVID-19 vaccine [6]. Furthermore, infection risks and vaccine safety were the main reasons for COVID-19 vaccine hesitancy among 341 pregnant and breastfeeding women in Qatar. The acceptance rate of the COVID-19 vaccine was higher in our study than in other reviewed studies, most probably due to the free vaccination and the enforcement policies in Saudi Arabia. The results showed that 74% of the participants had already received the vaccine, and 7% intended to take it, revealing a higher tendency among pregnant women in Saudi Arabia than in other countries. However, there was no statistically significant link between low socioeconomic status, specifically not having an income, with the tendency to take the vaccine, opposite to another study that showed an acceptance level below 45% in the United States, Australia, and Russia. In addition, data from Scotland showed that 15% of women who gave birth in August 2021 completed their vaccination, and only 23% of women aged 35-39 took two doses of the vaccine. While in the United Kingdom, less than one-third of pregnant women accepted the vaccine during pregnancy. Furthermore, there is a lower acceptance rate among younger women and women with lower socioeconomic status. In conclusion, the similarities between our study and former studies are the beliefs and worries of pregnant women regarding the harmful side effects on the fetus. The study has several strengths. A validated survey and a scientific worry scale were used to measure specific worries about taking the COVID-19 vaccine. In addition, the data source was MNG-HA hospital, which adds to the current literature on the patients' perspectives at public hospitals in Riyadh. However, there are a few caveats in our study. First, the population was taken from a single center/hospital, MNG-HA, in Riyadh. Therefore, the subjects shared similar characteristics creating selection bias and limiting generalizability. Second, due to governmental enforcement, most of the population has already received the COVID-19 vaccine. If the population included a majority of pregnant women who did not receive the vaccine, the results could be different, and the drivers of hesitancy could differ. Moreover, the cross-sectional nature of the study limits any causal inferences.

Recommendations

Based on the findings and conclusions stated, the following recommendations are proposed. Maternity healthcare professionals are highly encouraged to pay attention to the worries and concerns of their patients. Therefore, healthcare professionals play an essential role in raising awareness by educating pregnant women about the benefits and risks of not having the vaccine. On the other hand, public health professionals should plan and implement massive awareness campaigns to reassure pregnant women of the safety and importance of taking the COVID-19 vaccine and clarify the misinformation regarding the vaccine's impact on pregnant women and their babies as the pandemic is relatively new. In addition, it is expected to find a level of concern among the public, especially pregnant women, given the short time to invent these vaccines. Further studies should be conducted to understand and compare the status in different regions of Saudi Arabia. In addition, quantification of the monetary value generated by vaccine use in Saudi Arabia needs to be done in further research.

## Conclusions

This study showed a significant correlation between pregnant women's worries and the intention to take the vaccine. The concerns were mainly about the impact of the vaccine on themselves, their babies, and the pregnancy. However, we did not find a link between the level of education, the number of people in the household, the employment status, with the hesitancy to take the COVID-19 vaccine in pregnant women. Nevertheless, the study was essential to understand pregnant women's behavior and perceptions in Saudi Arabia and how to design future public health interventions considering their concerns. The rate of COVID-19 vaccine coverage among pregnant women in this study proved the strength and the success of the public health measures and government regulations implemented in Saudi Arabia during the COVID-19 pandemic, including prioritizing high-risk groups. Further studies are required to understand the drivers of hesitancy and acceptance of the COVID-19 vaccine among pregnant women across the different regions of the kingdom.
